# Whose bias gets coded? Psychology's role in decolonizing AI

**DOI:** 10.3389/fpsyg.2025.1657227

**Published:** 2025-11-03

**Authors:** Divya Lakshmi S, Visakh Mohan, P. S. Reeja, Elizabeth Alexander, Neethu Varghese, Alphonsa Kurian

**Affiliations:** ^1^Department of Computer Applications, Marian College Kuttikkanam Autonomous, Idukki, India; ^2^School of Social Work, Marian College Kuttikkanam Autonomous, Idukki, India; ^3^Department of Sociology, Bishop Chulaparambil Memorial College, Kottayam, India; ^4^PG Department of Social Work, KE College, Kottayam, India; ^5^Department of English, Bishop Chulaparambil Memorial College, Kottayam, India

**Keywords:** facial features, gendered competence bias, DSM-based distress, Western norms, Western-centric cultural representation, proxies for risk, risk profiling, gang affiliation

## Introduction

Who decides whose minds—and whose biases—get embedded in the algorithms that shape our world? The discipline of psychology, with its foundational assumptions and cultural biases, is deeply woven into artificial intelligence systems, influencing whose perspectives are valued and whose are marginalized. Bias in artificial intelligence is often described as a technical flaw, yet many of its origins are psychological—rooted in the constructs, methods, and epistemic values of psychology as a field. At the same time, bias also emerges from technical design choices, economic incentives, and governance structures. Our focus here is on psychology's distinctive role within this broader sociotechnical system (Ukanwa, 2024). This article argues that psychology both contributes to AI bias and holds unique potential to decolonize it through culturally inclusive design. To make this case, we first show how psychological constructs, often derived from Western, Educated, Industrialized, Rich, and Democratic (WEIRD) contexts, have been embedded into AI in ways that amplify inequities. We then draw on decolonial traditions in psychology to propose standards and practices that could steer AI toward global representativeness, epistemic justice, and liberatory outcomes.

Algorithms inherit ways of thinking that reflect Western-centric models of behavior and identity (Rodier et al., 2023). Foundational critiques in AI ethics have already shown that models risk reproducing and amplifying social and cultural hierarchies, for instance uncover in the influential “stochastic parrots” argument that large-scale models inevitably encode representational harms (Bender et al., 2021).

As machine learning models increasingly rely on psychological constructs such as decision-making, affect detection, and personality assessment in simulating or forecasting human behavior, such constructs—frequently borrowed from Western, Educated, Industrialized, Rich, and Democratic (WEIRD) societies—are universalized globally, often in spite of cultural specificity (Li et al., 2024; Peters and Carman, 2024). Recent empirical studies show that, in what ways language models possess not only linguistic but cultural and ideological biases as well, and we are forced to consider whose voices and perspectives they are representative of Santurkar et al. (2023). Such a procedure makes the AI technologies instruments for the communication not only of wisdom but ideological imperatives, and brings to the fore pertinent questions regarding whose psychology gets encoded and at whose expense (Shukla, 2025). Calls for more pluralistic models of AI alignment (Sorensen et al., 2024) are accurate where such a procedure exists, but a decolonial lens expands our eye to awareness that what gets encoded is not only behavior or cognitive processes but ideology, and brings with it the mandate for a psychological science realignment as a primary consideration in constructing ethically solidary, culturally inclusive technologies.

## Cognitive bias: psychology's double-edged sword

Psychology did much in teaching us about bias, from the initial work of Kahneman and Tversky on heuristics to the most recent on implicit bias (Dominguez-Catena et al., 2025). Although this research has informed contemporary AI development, it has not proved sufficient to prevent the emergence and propagation of harmful biases in AI systems. We recognize, however, that bias is not always inherently negative; as recent work shows, certain forms of bias can be benign or even beneficial, while others entrench inequities (Fabi and Hagendorff, 2022; Waters and Honenberger, 2025). Our focus in this article is on those biases that reproduce systemic disadvantage and cultural exclusion.

A classic example is in facial recognition technology employed in Detroit, where in 2023 the system misclassified Black faces five to 10 times more often than white faces (Basheer, 2024). The imbalance is traced to training sets dominated by Caucasian facial features and to psychological assumptions—such as the idea that emotions are universally expressed and recognized across all cultures—that do not account for cultural and phenotypic differences (Domnich and Anbarjafari, 2021). The National Institute of Standards and Technology confirmed these findings, reporting racial disparities in 189 algorithms developed by 99 different companies (Grother et al., 2019).

Similarly, Amazon's defunct recruitment algorithm penalized resumes containing keywords such as “women's chess club” or references to all-female colleges. The system reproduced historical gender biases embedded in its training data, effectively privileging male-coded language and experiences. While not a psychological mechanism *per se*, this outcome parallels attribution errors studied in psychology, where competence is inferred through biased cues, and illustrates how data-driven systems can replicate structural inequities (Mahapatra and Mujtaba, 2019; Venkateshwaran, 2025).

These cases illustrate how psychological constructs, when uncritically embedded into AI, can entrench and even amplify social inequities. Table 1 summarizes additional real-world examples of psychological constructs in AI systems and their resulting sociocultural impacts.

**Table 1:** Examples of psychological bias in AI and their impacts.

**Case study/Technology**	**Psychological construct embedded**	**Cultural/** **Demographic impacted**	**Key consequence/Harm**	**Citation(s)**
Detroit facial recognition	Universality of emotion/facial features	Black Americans	The system produced misidentification rates five to 10 times higher for Black individuals, resulting in wrongful arrests.	[Bibr B2][Bibr B6]; National Institute of Standards and Technology, 2022
Amazon recruitment algorithm	Gendered competence bias	Women applicants	Penalized resumes mentioning “women's,” underrepresentation	Mahapatra and Mujtaba, 2019; Venkateshwaran, 2025
Indian mental health chatbots (Wysa, Woebot)	DSM-based distress, Western norms	Indian users	Misinterpretation of local idioms, inappropriate recommendations	Viberg et al., 2023; Deva et al., 2025
Midjourney/DALL-E Generative AI	Western-centric cultural representation	Indian subcultures	73% poverty imagery for “Indian family dinner” prompts	Ghosh et al., 2024; Kumar et al., 2025
Dutch tax authority fraud algorithm	Proxies for risk (dual nationality, income)	Ethnic minorities	20,000 families wrongly accused, 1,000 children in foster care	Alba, 2024
Chicago predictive policing	Risk profiling, “gang affiliation”	Black neighborhoods	32% higher arrests despite equal crime rates	Khan and Ewuoso, 2024

## Standards for bias in AI

The preceding examples underscore how psychological constructs, when uncritically implemented, amplify inequities. But this raises a critical question: is all bias inherently harmful? Some scholars argue that the goal of “bias-free AI” is both unattainable and misleading, since all models reflect values, assumptions, and standpoints (Fabi and Hagendorff, 2022). The key issue is not whether bias exists, but whether it is reflexively acknowledged, equitably distributed, and aligned with principles of justice.

From a decolonial perspective, the distinction lies between biases that “punch down” (reinforcing domination and exclusion) and those that “punch up” (resisting oppression and amplifying marginalized voices). Decolonial psychology traditions—from Fanon's analyses of colonial subjectivity (Fanon, 1967) to Martín-Baró's liberation psychology (Martín-Baró, 1994) and Indigenous frameworks of relationality emphasizing interconnectedness and collective wellbeing (Wilson, 2008)—remind us that partiality can serve emancipatory ends when it surfaces silenced knowledge systems rather than suppressing them.

This article therefore rejects the ideal of “bias-free AI” in favor of a normative standard: AI systems must disclose their epistemic commitments, embrace cultural pluralism, and actively counter oppressive hierarchies. In practice, this means building technologies that recognize the inevitability of bias, while steering it toward inclusivity, accountability, and justice.

## Decolonizing psychology: the internal reckoning

The issue of bias in AI is not only technical but also connected to the long history of colonialism in psychology—pathologizing native cultures, medicalizing difference, and exporting Western diagnostic guidelines as if they were universal (Deva et al., 2025). Such epistemological biases are perpetually integrated into AI technologies. For example, the talk therapy conversational bots Wysa and Woebot, developed almost entirely from Western clinical databases, incorrectly interpreted culturally characteristic Indian expressions. A 2024 study identified that Indian users' statements such as “family pressure is my karma” were pathologized as depressive symptoms, deserving of unsuitable therapeutic interventions (Viberg et al., 2023). Generative models such as Midjourney and DALL-E also incorrectly represented Indian subcultures: a 2024 University of Pennsylvania study noted that the prompt “Indian family dinner” produced poverty-related images 73% of the time, while “American family dinner” produced middle-class settings.

These are described as psychological assumptions because they draw directly from psychological constructs—such as the universality of diagnostic categories (e.g., DSM-based criteria for distress), standardized measures of emotion, and Western notions of family and identity—that have historically been treated as objective and culture-free. When transferred into AI systems, such constructs enact a second-order colonization: exporting Western norms while erasing non-Western modalities of being under the guise of objectivity and universality (Ghosh et al., 2024; Kumar et al., 2025).

## Addressing injustice through decolonial AI practices

### Decolonial solutions in practice

Psychology can help repair these damages by fostering epistemic pluralism, co-design, and critical reflexivity. One strong example comes from South Africa, where researchers collaborated with Zulu communities to co-design a tuberculosis (TB) diagnosis algorithm. In Zulu, the idiom “isifuba sibuhlungu” literally means “the chest is painful,” but it carries cultural and linguistic nuances that early English-trained diagnostic systems failed to interpret correctly. Because these AI models were trained only on biomedical descriptions in English, they initially misclassified the condition, lowering diagnostic accuracy to 68%. By incorporating Zulu terminology and involving community members in the design process, accuracy improved dramatically to 92%. This shows that co-design not only raises technical precision but also affirms the legitimacy of local knowledge in medical AI.

There is a similar lesson from Aotearoa/New Zealand, in which Māori researchers established the Te Hiku voice model as an embodiment of the speaking voice of te reo Māori. Off-the-shelf commercial speech-recognition software had an error rate of as high as 47% among Māori speakers directly because they were excluded from international training datasets. By collating and controlling their own data sets, Māori communities brought errors to near zero while keeping their voices and the way they are stored in sovereign hands. The project proves that Indigenous-driven design is both feasible in terms of system performance increase and in terms of cultural sovereignty protection.

These exemplars conform with the new GPA guidelines (Global Psychology Alliance, 2023), that emphasize three practices: cultural disclosure, cross-validation, and site-based audits. Cultural disclosure obliges researchers to be transparent in marking the cultural heritage of their data so that Western constructs will cease being presented as universal. Cross-validation over three or more settings demands that psychological constructs and AI models be proved in diverse cultural settings rather than in WEIRD populations so that they can be applied more widely. Site-based audits demand independent, often community-based examination of psychological research and applications of AI so that ethical propriety as well as cultural sensibility may be established.

Applied to AI, these standards mean that therapeutic chatbots, diagnostic algorithms, or predictive models should (1) disclose the cultural provenance of their training data, (2) undergo validation in multiple culturally distinct populations, and (3) be audited by independent panels that include community stakeholders. Together, these measures shift psychology from passive collaborator in bias to active ethical design architect, embedding justice and cultural pluralism into the development of AI (Masaka, 2019; Ofosu-Asare, 2024).

## Rewriting the code of psychology

The question of whose bias gets coded necessarily opens onto the complicity of psychology in the empowerment of systems that disguise ideology as objectivity. The examples—from the Dutch tax algorithm's racial bias through Chicago's predictive policing—are used here just to illustrate how psychological inventions, disguised under the pretense of universal laws, have themselves been used to encode the colonial relations of power in AI. Not the predestined product of technology but the product of political and disciplinary choices, they may be undone through the decentralized production of knowledge through both cross-cultural validation as well as community co-design. Cross-cultural validation and community co-design are related but distinct processes: validation demonstrates that a model's performance is not WEIRD-restricted, while co-design ensures the model is legitimate, safe, and valuable in a specific cultural setting. Tensions will inevitably arise—for example, when a community prefers a design that is not yet cross-culturally validated, or when a validated system conflicts with local norms. In such cases, we recommend a dual-criterion protocol weighing generalizability against local acceptability: deployments are only to proceed when both a generalizability threshold (G) and a locally set acceptability threshold (L) are met. Where threshold values are different, deployment is either to be limited to local scale with explicit labeling (L≥L^*^, G < G^*^) or deferred until redesign and governance protection are in place (G≥G^*^, L < L^*^). It thus makes explicit why co-design is necessary, in what respects it is different from validation, and in what respects such conflicts are responsibly to resolve.

Epistemic responsibility must draw sustenance also from independent data and algorithmic audits. Such audits must take place under the aegis of cross-disciplinary working groups of academic specialists, civic societies, and citizens from communities most at risk of the effects of AI. Their remit is in technical proficiency as well as in representational democracy, such that fairness, cultural compatibility, and transparency must be taken into account in many different lexicons. Justice must become an organizing principle both in the generation and in use of AI. Psychology is at a fork in the road at present: it can become an architecture of exclusion, or become the scaffold for liberation through the generation of culturally compatible and ethically informed systems of AI. The code, and the effects of code, is ours to rewrite.
